# Location of Moving Targets in Substation Non-Line-of-Sight Environment

**DOI:** 10.3390/s19235321

**Published:** 2019-12-03

**Authors:** Yubo Wang, Weimin Yang, Zheng Wang, Wenjun Zhou, Liang Li, Hongsen Zou

**Affiliations:** 1 State Grid Key Laboratory of Power Industrial Chip Design and Analysis Technology, Beijing Smart-Chip Microelectronics Technology Co., Ltd., Beijing 100192, China; wangyubo@sgitg.sgcc.com.cn (Y.W.); wangzheng3@sgitg.sgcc.com.cn (Z.W.); liliangbj@163.com (L.L.); 2 School of Electrical Engineering and Automation, Wuhan University, Wuhan 430072, China; ywm@whu.edu.cn; 3 State Grid Ningxia Electric Power Co., Ltd., Yinchuan 750011, China; zouhongsen@nx.sgcc.com.cn

**Keywords:** substation, wireless sensor network (WSN), moving target, non-line-of-sight (NLOS), probability distribution

## Abstract

In substations, a localization system based on a wireless sensor network (WSN) is a challenge, because the propagation of the measured signal could be blocked by various devices. In other words, non-line-of-sight (NLOS) propagation, where the signal propagation path is occluded, will affect measurement accuracy. A novel localization method based on a two-step weighted least squares and a probability distribution function is proposed to reduce the influence of NLOS error on the localization result. In this method, the initial multi-group localization result is obtained by the two-step weight weighted least-squares method, and the probability distribution function of the target is constructed by using the initial localization results, which can effectively reduce the influence of the NLOS error on the localization result. The simulation and test results show that the proposed method can keep the coordinate error within 30 cm in the substation. Compared with the localization result of two-step weighted least-squares (TSWLS) method, the average localization error is reduced by more than 1 m. Compared with the other two similar algorithms, the localization accuracy is improved by more than 50%. The tested results show that the localization performance of the method is robustness in the NLOS environment of the substation. While ensuring stability, the proposed algorithm is less efficient than some existing ones. However, under the calculation conditions of ordinary computers, the average single-point calculation time is less than 0.1 s, which can meet the needs of applications in substations.

## 1. Introduction

In power substations, power equipment is densely populated. Due to the high level of voltage, there are many high-risk working areas. Inadvertent entry of moving targets such as inspectors and equipment may result in loss of life and property [1,2]. Currently, there are no reliable technical means and measures to protect human life in power substations. The safety of substation staff is mainly reliant on the consciousness of the safety supervisors, which is time-consuming, laborious, and subjective. During commissioning and maintenance work of substations and transmission lines, it is often necessary to use tall vehicles, such as large cranes. When a work vehicle works near live equipment or under live transmission lines, personnel supervision is usually required to prevent the working vehicle from touching the line. As there may be personnel fatigue, or care may not be timely, and the safety risks are high. If the location of the moving target in a substation can be obtained, and an alarm may be used to promptly warn the personnel involved, the safety of the staff can be guaranteed. In recent years, ultra-wide band (UWB) technology, which is a wireless carrier communication technology, has been used in the field of moving target localization. The UWB signal has a frequency range of 3.1 GHz to 10.6 GHz, and a bandwidth exceeding 500 MHz. It has a transmit power of less than −41 dBm/MHz. The UWB technology enjoys a good anti-multipath performance, good penetration capability, and a low power consumption. By constructing a wireless sensor network (WSN) based on UWB technology [3,4], the base station can obtain distance information between the target to be located and the base station by analyzing the UWB signal transmitted by the tag carried on the target. How to use the distance information to accurately estimate the position of the moving target is of great significance.

The location estimation of unknown targets is a typical nonlinear estimation problem, which is challenging. To achieve accurate localization of moving targets, scholars conducted a lot of research and proposed many classic localization algorithms, such as weighted least squares (WLS) [5,6], maximum likelihood (ML) estimation [7,8], and so on. These algorithms can achieve better localization accuracy under line-of-sight (LOS) conditions. However, in the non-line-of-sight (NLOS) [9] environment, where the signal propagation path is occluded, the performance of these algorithms usually drops. Therefore, to achieve accurate localization in the NLOS environment, it is often necessary to introduce some mechanisms to reduce the negative impact of NLOS error on the localization algorithm. In recent years, some localization algorithms have been developed to improve accuracy in the cases of NLOS propagation. In [10,11,12], algorithms can filter out unnecessary measurement information in situations of NLOS propagation, and retaining that essential measurement information only for localization. However, in an environment with many NLOS signals, there will be a situation in which the amount of data that are reliable is too small. As a result, the localization accuracy may be seriously affected. Reference [13] points out that in a propagation environment with mixed LOS/NLOS, making good use of NLOS measurement information can improve the accuracy of position estimation. In [14,15,16], the error distribution information of the measured signal in space was obtained in advance, and then the measurement result, which is identified as NLOS measurement, will be reconstructed to reduce the influence of the NLOS measurement error. Such methods need to obtain a priori information of the NLOS signal in the environment [17]. In addition to the methods in the literature [14,15,16], there is a class of method [18,19,20,21] that obtains the final localization result by weighting all measurement information. However, the weights in such methods are often calculated from a priori information measured in the field. As the error distribution of the NLOS measurement typically has much more complex properties and cannot be captured by a simple model [22], this type of localization methods is difficult to stably and accurately locate in a complex environment such as a substation.

A class of probabilistic location methods based on information fusion has been reported. The Kalman filter (KF) [23,24,25]-based method and the particle filter (PF) [26]-based methods are the most widely used. The main problem of the Kalman filter-based localization algorithm is that both the system noise and the perceptual assumed noise are subject to Gaussian distribution. In [27], the method of reconstructing the error matrix is used to reduce the error distribution requirements of the localization algorithm. The particle filter-based localization algorithm is suitable for nonlinear non-Gaussian localization systems. The main problem is particle depletion and computational complexity. References [28,29] proposed different schemes to reduce the number of particles required by the PF, which improved computational efficiency while ensuring location robustness. In general, KF-based localization algorithms and PF-based localization algorithms can achieve accurate and stable localization. However, such localization algorithms usually require different types of measurement data to establish a state model of the localization target, thereby realizing the establishment of the posterior probability distribution of the object. Although the localization algorithm based on KF and PF can correct the localization trajectory in the localization system with only the ranging sensor. In the localization system with only the ranging sensor, the robustness of the KF and PF-based localization algorithms may be degraded. When the motion state of the object changes, the localization accuracy of the algorithm will be affected. Therefore, KF-based localization algorithms and PF-based localization algorithms are usually applied to localization systems with multiple sensors for measurement, such as the localization of robots that integrate targets of various sensors. For mobile targets such as mobile workers or mobile vehicles in a substation, to facilitate carrying and controlling costs, only the distance-sensing tags are often carried. Therefore, it is necessary to study a localization algorithm that can achieve better robustness and accuracy under the condition that only distance measurement information can be achieved.

To improve the robustness and accuracy of the localization algorithm when using only ranging information, this paper proposes a new method based on probability distribution function. The method proposed in this paper does not need to identify the NLOS measurement, nor does it require a priori knowledge of the distribution or statistics of the NLOS errors. All it needs is the LOS measurement error distribution. The difference between this method and the probabilistic methods based on PF or KF is that the proposed method is to construct the error distribution function by using the excess distance sensor data in the localization system and the KF and PF algorithms are localization algorithms that establish the probability of object distribution based on the position and motion state information of the object. The proposed algorithm has almost no dependence on the motion state information, and can ensure accuracy and robustness in the localization system with only the distance sensor. On the other hand, in contrast to the existing localization methods that use the NLOS prior error distribution to correct the results, the localization algorithm proposed in this paper uses the measured data acquired in real time to construct the probability distribution function of the localization target in space, and then achieve the position estimation of the target. By grouping and initializing a large number of real-time ranging data, multiple possible coordinate points are obtained, and the error probability distribution function is constructed according to these points, which can effectively improve the robustness and accuracy of the algorithm, which is an important contribution of this paper.

The detailed procedure of the proposed algorithm can be summarized as follows. First, to make a brief screening of the base station to ensure the computational efficiency of the algorithm, a simple base station selection principle is proposed, which can improve the calculation speed of the algorithm while ensuring the localization accuracy. This is one of the important contributions of this article. Secondly, all the data that participate in the localization are grouped, and each group of data is used for initial localization. Finally, the probability distribution function of the target is constructed by using multiple sets of initial localization results, and the target coordinates are obtained. This method can effectively improve the robustness of the algorithm and reduce the influence of measurement error on the localization result. This is another important contribution of this paper. The localization performance of the proposed algorithm is examined by simulation and experiment.

This paper is organized as follows. In Section 2, we formulate the localization model and method. In Section 3, the simulation is carried out, and the results are discussed. The field test and analysis are introduced in Section 4. Section 5 is the conclusion of this paper.

## 2. Localization Method Based on Two-Step Weighted Least Squares and Probability Distribution

### 2.1. Time Difference of Arrival (TDOA) Localization Model

The time difference of arrival (TDOA) [30,31] is a method for target localization based on analysis of the time difference of signals associated with the target. To achieve spatial localization, at least the time difference between the target and 4 different localization base stations should be known. However, when there are only 4 base stations for localization, there is a great possibility of large errors. Therefore, in actual localization applications, it is often ensured that the number of base stations is more than four.

It is assumed that a base station of *M* (*M* > 4) with known positions is set in the three-dimensional space, and the coordinates of the *i*-th base station are expressed as ***S_i_*** = (*x_i_*,*y_i_*,*z_i_*)^T^, *i* = 1, 2, …, *M*. The coordinates of the target are expressed as ***U*** = (*x*,*y*,*z*)^T^, and the distance from the target to the *i*-th base station can then be expressed as:

(1) $$r_{i}=\sqrt{{\left(x_{i}-x\right)}^{2}+{\left(y_{i}-y\right)}^{2}+(z_{i}-z{)}^{2}}$$

The first base station ***S*_1_** is selected as the reference base station for TDOA localization, and the TDOA measurement value can be expressed as:

(2) $$\tau_{i1}=t_{i1}+\Delta t_{i1}=(r_{i}-r_{1})/c+\Delta t_{i1},\;i=2,3,\ldots ,M$$

in (2), *τ*_*i*1_ represents the time difference measurement value between the measurement target and the base station ***S_i_*** and ***S*_1_**, *t_i_* and *t_1_* are the time values at which the signal arrives at the base station ***S_i_*** and ***S*_1_**. Δ*t*_*i*1_ is the measurement error of time difference between ***S_i_*** and ***S*_1_**, and *c* is the speed at which electromagnetic waves propagate in a vacuum.

By multiplying the two sides of (2) by the speed of light, the equation below can be obtained:

(3) $$d_{i1}=c\tau_{i1}=r_{i1}+n_{i1}=r_{i}-r_{1}+n_{i1},\;i=2,3,\ldots ,M$$

in (3), *r*_*i*1_ = *r_i_* − *r*_1_ is the distance between the measurement signals reaching the two base stations, and *n*_*i*1_ = cΔ*t*_*i*1_ represents the measurement error of the difference in their distance to the base station. By combining (3) and (1), the localization equation can be obtained:

(4) $$d_{i1}=\sqrt{{\left(x_{i}-x\right)}^{2}+{\left(y_{i}-y\right)}^{2}+(z_{i}-z{)}^{2}}-\sqrt{{\left(x_{1}-x\right)}^{2}+{\left(y_{1}-y\right)}^{2}+(z_{1}-z{)}^{2}}+n_{i1}$$

where *i* = 2, 3, …, *M*. Without considering the NLOS error, it can be assumed that the measurement error *n*_*i*1_ in (4) obeys a normal distribution with a mean of 0 and a variance of *σ*. By solving the optimal estimate of (4), the coordinates of the location of the target can be located.

### 2.2. Initial Localization Method Based on Two-Step Weighted Least Squares

To obtain a more effective localization algorithm, Equation (4) is transformed into a linear equation, and then the optimal estimation is obtained by the two-step weighted least-squares (TSWLS) [6] method as the initial localization result.

Simultaneously squaring the two sides of Formula (3), we can obtain:

(5) $$[(d_{i1}-n_{i1})+r_{1}{]}^{2}={\left(r_{i1}+r_{1}\right)}^{2}=r_{i}{}^{2}$$

Then substitute Equation (1) into Equation (5).

(6) $${\left[(d_{i1}-n_{i1})+r_{1}\right]}^{2}={x_{i}}^{2}+{y_{i}}^{2}+{z_{i}}^{2}-2x_{i}x-2y_{i}y-2z_{i}z+x^{2}+y^{2}+z^{2}$$

And we can express Equation (6) in the form of a vector:

(7) $${\left[(d_{i1}-n_{i1})+r_{1}\right]}^{2}=S_{i}{}^{T}S_{i}-2S_{i}{}^{T}U+x^{2}+y^{2}+z^{2}$$

Subtracting both sides of Equation (7) by *r*_1_^2^, we can get:

(8) $${d_{i1}}^{2}-2d_{i1}n_{i1}+{n_{i1}}^{2}+2r_{1}(d_{i1}-n_{i1})=S_{i}{}^{T}S_{i}-S_{1}{}^{T}S_{1}-2(S_{i}-S_{1}){}^{T}U$$

Sort out the two sides of Equation (8), we can get:

(9) $$0.5\times ({d_{i1}}^{2}-S_{i}{}^{T}S_{i}+S_{1}{}^{T}S_{1})+(S_{i}-S_{1}){}^{T}U+d_{i1}r_{1}=(r_{1}+d_{i1})n_{i1}=(r_{1}+r_{i1})n_{i1}+{n_{i1}}^{2}$$

Ignoring the quadratic term of the error term in Equation (9) and organize it into a matrix form:

(10) 0.5×h−Gφ=η

where

$$h=\left[\begin{array}{c} d_{21}^{2}-S_{2}^{T}S_{2}+S_{1}^{T}S_{1}\\ \vdots \\ d_{M1}^{2}-S_{M}^{T}S_{M}+S_{1}^{T}S_{1} \end{array}\right],\;G=[\begin{array}{cc} {\left(S_{1}-S_{2}\right)}^{T} & -d_{21}\\ \vdots & \vdots \\ {\left(S_{1}-S_{M}\right)}^{T} & -d_{M1} \end{array}],\;\varphi =\left[\begin{array}{c} U\\ r_{1} \end{array}\right],$$

$$\eta =Bn,\;B=\mathrm{diag}\{r_{2},\cdots ,r_{M}\},\;n=[n_{21},\cdots ,n_{M1}]$$

Equation (10) is linearized from a nonlinear equation to a linear equation. We can get the coordinates ***U*** of the target from ***φ***. The least-squares (LS) method can be used to obtain the best estimate of ***φ*** :

(11) $$\varphi =\arg\;\min\{{\left(0.5*h-G\varphi \right)}^{T}\Psi^{-1}(0.5*h-G\varphi )\}={\left(G^{T}\Psi^{-1}G\right)}^{-1}G^{T}\Psi^{-1}h$$

Here ***Ψ*** is the weighting matrix of Equation (11), ***Ψ*** = *E* (***ηη***^T^) = *c*^2^***BQB***, where ***Q*** is a diagonal matrix with a diagonal element of 0.5*σ*^2^. It represents an error covariance matrix. ***Ψ*** is not known in practice as ***B*** contains the true distances between source and receivers. Further approximation is necessary to solve the problem. According to the fact that the difference between the two sides of the triangle is smaller than the third side, we know that *r_i_*−*r*_2_ is less than the distance between the two sensors. The distance between the sensors is fixed. When the distance of the localization target distance sensor is greater than 20 times the distance between the sensors, the difference between any two *r_i_* will be less than 5% of the corresponding *r_i_*, and it can be seen that each *r_i_* is equal, so we can assume that ***B*** ≈ *r*_2_***I***, ***I*** is an identity matrix. Equation (11) can be approximated as:

(12) $$\varphi \approx {\left(G^{T}Q^{-1}G\right)}^{-1}G^{T}Q^{-1}h$$

And then an estimated value of *r*_1_ can be obtained by using Equation (12). After that we can use *r*_1_ to calculate the estimated value of *r_i_*. From which matrix ***B*** can be obtained. Then matrix ***B*** can be substituted into Equation (11) to obtain the position estimation result.

When using (11) to estimate the position of the target, in a LOS condition, the root mean squared error (RMSE) of the localization result can be close to the Cramer–Rao bound [32], and the calculation is very simple. With the increase of the measured value, the two-step LS method can use the measurement data of the redundant base station to improve the localization accuracy. However, in the case of a large LOS measurement error or NLOS propagation, the localization accuracy of the two-step linear LS method cannot be guaranteed. Consequently, one must perform the initial solution of Equation (10). To achieve precise localization in the complex environment of substation, the results need to be further optimized based on the initial estimation. Here, a probability distribution function is introduced to further improve the position estimation.

### 2.3. Location Estimation Algorithm Based on Probability Distribution Function

In the substation environment, the propagation path of a measurement signal includes both the LOS path and the NLOS path. The measurement error under the LOS path is small, and is generally regarded as due to white noise obeying a normal distribution. The propagation error under the NLOS path is associated with the propagation environment. Without the propagation error, the aforementioned equations can be solved with a unique optimal solution. However, the LOS error and NLOS error existing in the measured value make it difficult for the numerical method to converge to the optimal solution when solving the equation. From a statistical point of view, the localization result obtained by the combination of the data with higher LOS measurement value is closer to the actual coordinate of the target, and the localization result is consistent and obeys the spatial normal distribution. Localization results that are more affected by the NLOS measurement data will deviate from the region and will not exhibit a regular distribution. Therefore, the concept of spatial probability distribution is introduced. Through the distribution of most of the localization results that are less affected by the NLOS measurement data, the target will have the smallest probability of error in space. Localization results with large errors will deviate from the region, do not have uniform regularity, and will not affect the probability distribution of the region.

Assuming that the number of base stations is *N* > 4, the number of measurement values that can be obtained is *N* − 1. In this paper, data combination refers to a data set containing some measurement data. Group all the measurement values, *N* − 1 measurement values can be expressed as $C_{N-1}^{4}$ which is a data combination containing 4 measured values. For each data combination, one set of localization results can be obtained. Due to the LOS error and the NLOS error, the localization results of each data combination are not the same, and there is an error among the calculated coordinates and the actual coordinates.

Reference [33] proposes that when the TDOA time difference measurement error is large, in the polar coordinate system, the directional angle information calculated by the localization algorithm is more accurate than the modular length information calculated by the localization algorithm. Therefore, after considering the localization result as a spatial straight line, the more accurate direction information can be used to obtain the final result.

Suppose there are *U* data combinations (O_1_, O_2_, …, O*_j_*, …, O*_U_*), where *j* = 1, …, *U*. We solve each data combination separately, and record the result coordinates (*x_pj_*, *y_pj_*, *z_pj_*). At the same time, the geometric center of all the base stations in each data combination is recorded, its coordinates are expressed as (*x*_O*j*_, *y*_O*j*_, *z*_O*j*_). The two points of the known coordinates in the space form a straight line. Using the data combination initial localization result (*x_pj_*, *y_pj_*, *z_pj_*) and the data combination geometric center (*x*_O*j*_, *y*_O*j*_, *z*_O*j*_), a space line can be established using (13).

(13) $$\left\{ \begin{array}{l} \frac{x-x_{Oj}}{\cos\theta_{Oj}}=\frac{y-y_{Oj}}{\sin\theta_{Oj}}=\frac{z-z_{Oj}}{\tan\phi_{Oj}}\\ \theta_{Oj}=\arctan\frac{y_{pj}-y_{Oj}}{x_{pj}-x_{Oj}}\\ \phi_{Oj}=\arctan\frac{z_{pj}-z_{Oj}}{\sqrt{{\left(x_{pj}-x_{Oj}\right)}^{2}+{\left(y_{pj}-y_{Oj}\right)}^{2}}} \end{array}\right.$$

The probability *p_j_*(*θ*,*ϕ*) of any point in the space is the unknown target satisfying (14), where *σ_θ_* and *σ_ϕ_* are the root mean square errors of the LOS measurement error after conversion to azimuth.

(14) $$p_{j}(\theta ,\phi )=1-\frac{1}{\sqrt{2\pi }\sigma_{\theta }\sigma_{\phi }}\exp\left\{-\frac{1}{2}[\frac{{\left(\theta -\theta_{Oj}\right)}^{2}}{2\sigma_{\theta }^{2}}+\frac{{\left(\phi -\phi_{Oj}\right)}^{2}}{2\sigma_{\phi }^{2}}]\right\}$$

Equation (15) is the joint probability density of each direction vector, and the point with the smallest joint probability density in space is the target coordinate to be located.

(15) $$p(\theta ,\phi )=\prod_{j=1}^{U}p_{j}(\theta ,\phi )$$

In (15), *θ* and *Φ* respectively represent the direction angle of the standard point (*x*_O*j*_, *y*_O*j*_, *z*_O*j*_) of any point in the space and the corresponding data, as shown in (16):

(16) $$\left\{ \begin{array}{l} \theta =\arctan\frac{y-y_{Oj}}{x-x_{Oj}}\\ \phi =\arctan\frac{z-z_{Oj}}{\sqrt{{\left(x-x_{Oj}\right)}^{2}+{\left(y-y_{Oj}\right)}^{2}}} \end{array}\right.$$

After calculating (15), the joint probability density of each point in space can be normalized according to Formula (17), and the probability distribution of the target in a certain region can be obtained. The larger the value of Formula (17), the greater the probability that the localization result exists in the space enclosed by the three-dimensional surface.

(17) $$p(x,y,z)=p(\theta ,\phi )=1-\frac{p(\theta ,\phi )-\min(p(\theta ,\phi ))}{\max(p(\theta ,\phi ))-\min(p(\theta ,\phi ))}$$

By using the error distribution range of the localization result, it is possible to locate more effectively, and to avoid unnecessary accidents caused by the deviation of the localization coordinates.

When passing the equation to the spatial probability density function of (14), it is necessary to know the root mean square error of the azimuth angle *θ_j_* and the pitch angle *ϕ_j_* due to the device measurement error under the LOS condition. It was obtained by experimental statistics in this paper. The experimental method is: (i) placing the localization tag at a known coordinate position of the substation; (ii) using the previous method to detect the tag at different positions of the substation; and (iii) using the two-step linear LS method to calculate the azimuth angle *θ_j_*, *ϕ_j_*. Finally, in (iv), using (18) to calculate the root mean square error, where *n* is the number of experiments and *θ*_ave_ is the average of *n* experimental measurements *θ_j_*. A total of 50 experiments were performed.

(18) $$\sigma_{\theta }=\sqrt{\frac{1}{n}\sum_{j=1}^{n}{\left(\theta_{j}-\theta_{\mathrm{ave}}\right)}^{2}}$$

### 2.4. Base Station Selection

The probability distribution-based localization method requires data from all the base stations to locate the target. When constructing the probability distribution function, the $C_{N-1}^{4}$ group data should be solved. Because of the increase of *N*, the calculation amount of the algorithm is multiplied. When *N* < 10, the number of data combinations does not exceed 100. Therefore, the upper limit of the number of base stations that participate in localization is set to 9.

There are two main principles for selecting a base station. The first is to select the base station which presents LOS propagation conditions. The second is to choose those base stations that are that are closer to the target after the consideration of the first principle.

Since the difference between the two sides of the triangle is smaller than the third side in length, the distance difference between the target of the two base stations is smaller than the distance between the two base stations. However, if there exists NLOS propagation when a localization signal reaches a certain base station, the time for the localization signal to reach the base station is greatly increased. Generally, the base station whose signal arrives at the earliest time is selected as the reference base station. The measured value *d*_*i*1_ of the distance difference between the *i*-th base station and the reference base station is obtained using Equations (2) and (3). The distance *D*_*i*1_ between the base stations can be calculated from the known base station coordinates. If *d*_*i*1_ ≥ *D*_*i*1_, it can be determined that there is NLOS propagation between the target and the *i*-th base station. Therefore, the primary screening of all the base stations can remove the localization base stations that are greatly affected by the NLOS propagation.

After screening through the above steps, the remaining base stations can be used for localization. If the number of remaining base stations is still greater than 9, the 9 base stations with the earliest arrival time of the signal are selected to participate in the localization. When the number of remaining base stations is less than 4, the 3 base stations with the earliest signal arrival time are selected among the excluded base stations to participate in the localization.

### 2.5. Steps of the Localization Method

**Figure 1:**
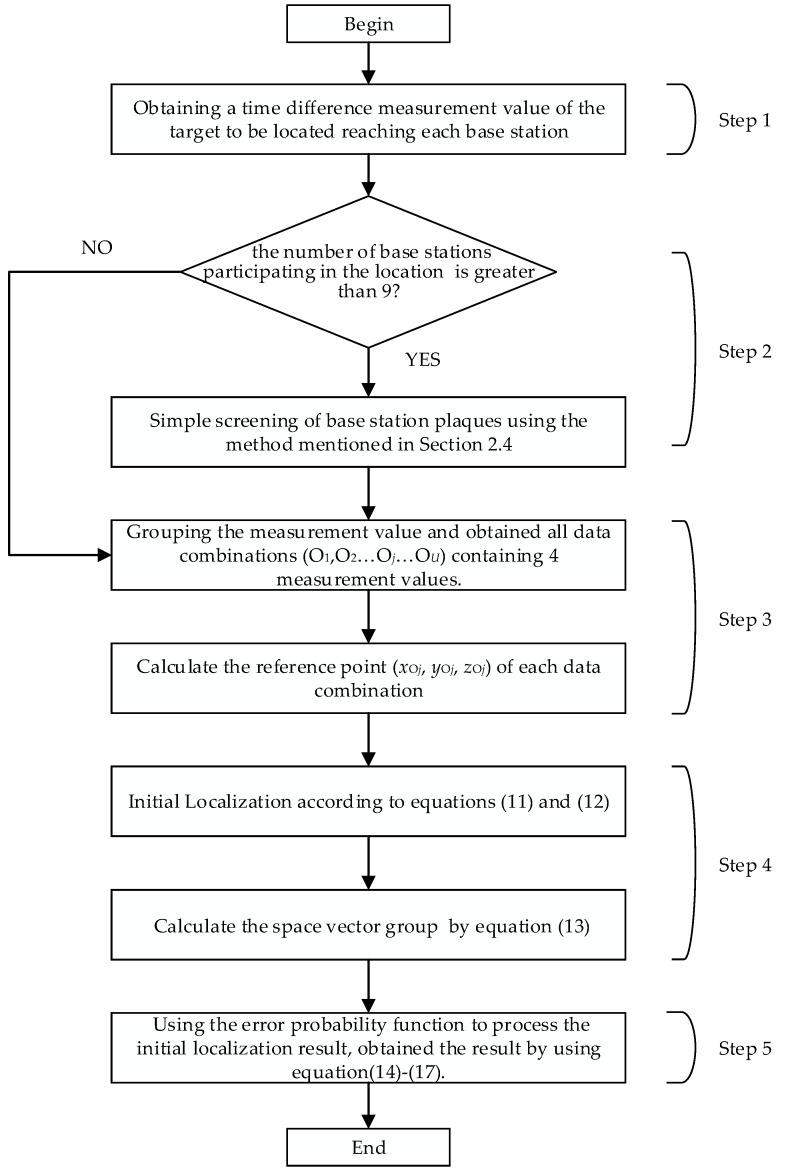
The flow chart of the proposed method.

A novel localization method based on TSWLS and probability distribution function is adopted here. The specific flow chart of the proposed method is shown in Figure 1, which can be further elaborated as follows:

Step 1: A coordinate system is established in the substation, and the localization base station is arranged at the designated position. Obtaining the time difference between the signal emanated from the target to be located and each base station

Step 2: Screening the base station. When the number of base stations participating in the location of the target is greater than 9, the appropriate localization base station is selected by using the procedure described in Section 2.4. When the number of base stations is less than or equal to 9, all base stations are selected as the localization base station;

Step 3: Grouping of measured values. From the *N* (*N* > 4) localization base stations obtain *N* − 1 measurement values as described earlier. Formulate $C_{N-1}^{4}$ data combinations (O_1_, O_2_, …, O*_j_*, …, O*_U_*) containing four measured values. Then calculate the reference point (*x*_O*j*_, *y*_O*j*_, *z*_O*j*_) of each data combination;

Step 4: Initial Localization. According to Equation (11) and Equation (12), the position estimates of the different data combinations are obtained. The solution of the equations can be expressed as (*x_pj_*, *y_pj_*, *z_pj_*), and the space vector group is established by Equation (13).

Step 5: Using the probability distribution function to process the initial localization result. The spatial probability distribution model is established by Equations (14)–(17), and the point where the error probability in space is the smallest is set as the coordinate of the target to be located.

## 3. Simulation

To verify the localization performance of the proposed algorithm, this section carries out a simulation study on the 3 existing algorithms, namely the algorithm proposed in the paper, the TSWLS [6] algorithm, residual weighted (RWGH) [34] algorithm, and the residual test (RT) [17] algorithm. The two-step WLS algorithm uses the LS method to estimate the position of the target, and is generally used for localization under LOS conditions. The RWGH algorithm weights different results by the residual of the initial localization result to reduce the influence of the NLOS error. The RT algorithm compares the calculated residual with a set threshold to identify the NLOS base station in the environment. Then, by removing the measurement data obtained by the NLOS base station, the purpose of improving the accuracy of the algorithm is achieved.

The simulation results are compared and analyzed to verify the superiority of the proposed algorithm.

### 3.1. Simulation Parameters

**Figure 2:**
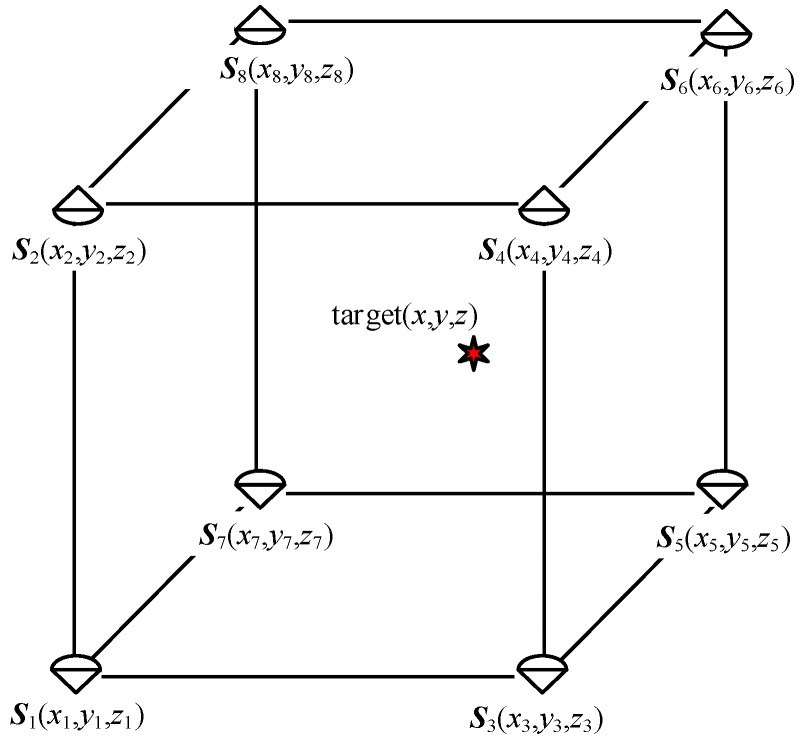
Schematic diagram of the simulation environment space.

The simulation conditions are set as follows: The targets to be positioned were randomly distributed in a space area of 30 m × 30 m × 3 m. There were 8 base stations in the to-be-positioned area, and the coordinates were respectively as ***S*_1_** = (15,15,0) ^T^, ***S*_2_** = (−15,15,0) ^T^, ***S*_3_** = (15, −15, 0) ^T^, ***S*_4_** = (−15, −15, 0) ^T^, ***S*_5_** = (15, 15, 3) ^T^, ***S*_6_** = (15, 15, 3) ^T^, ***S*_7_** = (10, 10, 3) ^T^, ***S*_8_** = (10, 10, 3) ^T^. The simulation environment is shown in Figure 2.

In the simulation, the LOS measurement error obeys a normal distribution with the mean value of 0, the standard deviation of σ. The NLOS error obeys the uniform distribution *b_i_*~*U* (0, *B*), where *B* is the maximum possible value of NLOS error. The simulations in this section were based on the Monte Carlo simulation method, and the number of Monte Carlo experiments is 2000, i.e., the average value of the localization error was obtained by randomly taking 2000 points in the space to be located for localization.

To verify the localization performance of the algorithm under different conditions, this section sets four different simulation conditions. The simulation conditions are set as shown in Table 1.

Simulation 1 compared the performance of the algorithm with the LOS measurement error standard deviation in the absence of NLOS measurement error, which reflect the relationship between the performance of the algorithm and the performance of the localization sensor. Simulation 2 and Simulation 3 compared the performance of the four algorithms with the NLOS measurement error, and reflected the relationship between the performance of the algorithm and the propagation environment, respectively. Simulation 2 simulated the situation when the maximum possible error of NLOS changes, and simulation 3 simulated the situation when the number of NLOS base station changes. The evaluation index of the performance of the algorithm from Simulation 1 to Simulation 3 was the RMSE of the position estimate and the true value. Simulation 4 further tested the performance of several algorithms. The cumulative distribution function (CDF) was chosen to compare these four algorithms.

### 3.2. Simulation Result and Discussion

**Figure 3:**
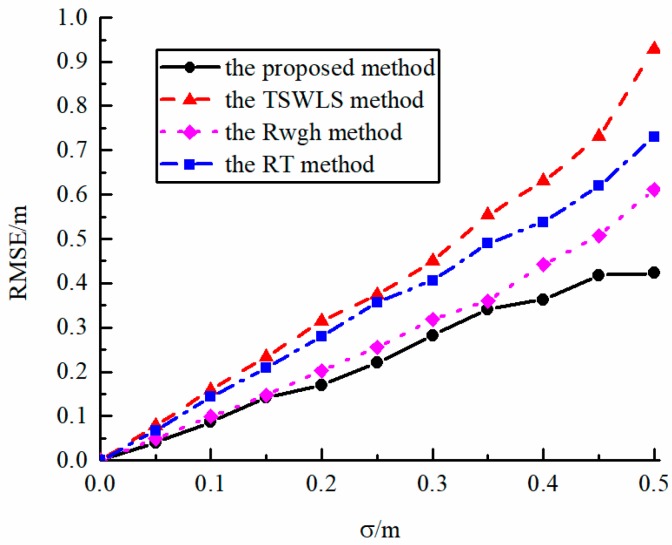
The error curves of various algorithms in LOS environment.

Figure 3 shows the relationship between the performance of the algorithm and the standard deviation σ of the LOS measurement error without NLOS measurement error. It can be seen from the figure that the localization performance of each algorithm is similar when the measurement error is small. However, when the standard deviation σ is high and the localization sensor performance is poor, the root mean square error of the localization algorithm proposed in this paper is significantly smaller than other algorithms, and it has better localization stability.

**Figure 4:**
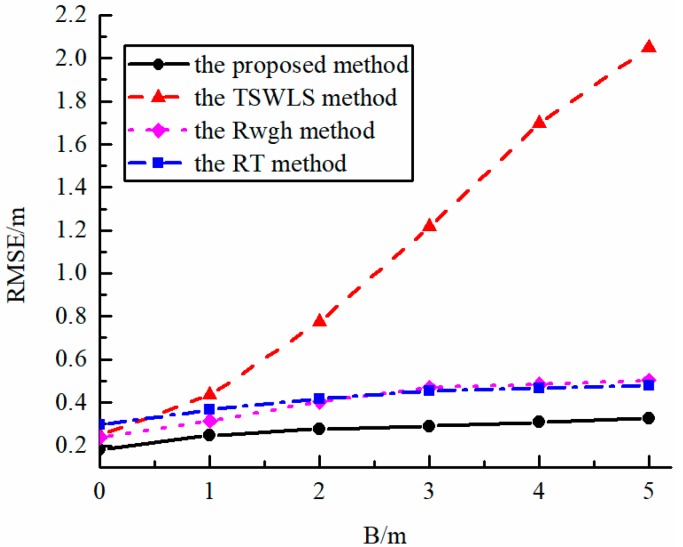
The error curve of various algorithm when NLOS error changes (2 NLOS base stations).

Figure 4 shows the error curve of the algorithms with their performances as a function of NLOS measurement error when several other conditions are the same. It can be seen from the figure that the TSWLS algorithm failed to maintain a good localization performance in the LOS/NLOS mixed propagation environment, while the RWGH algorithm, the RT algorithm and the proposed algorithm can reduce the influence of NLOS signal on the localization result in this mixed propagation environment. When the NLOS error is large, the proposed algorithm has better localization performance.

**Figure 5:**
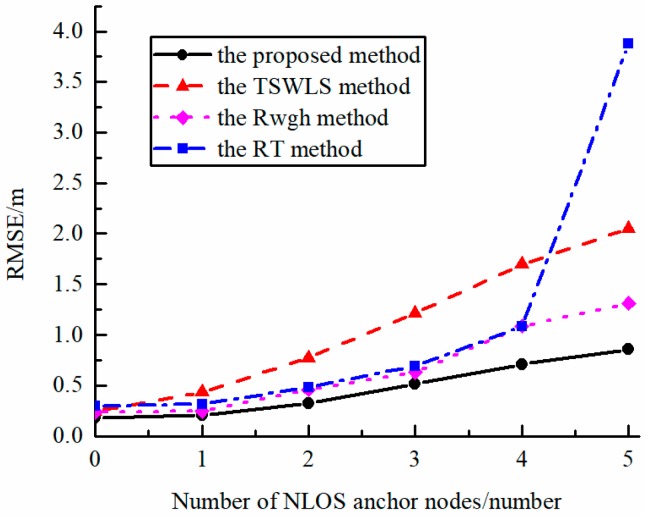
The error curve of various algorithm performance when the number of NLOS base stations changes (the maximum error of NLOS is 3 m).

Figure 5 shows the relationship between algorithm performance and the number of NLOS base stations. It can be seen from the figure that as the number of NLOS base stations increased, the localization performance of all algorithms decreased. The localization algorithm based on residual identification sharply declined in localization performance when half of the base stations were changed to NLOS ones. The proposed algorithm maintained high localization accuracy when there were large proportion of NLOS base stations.

**Figure 6:**
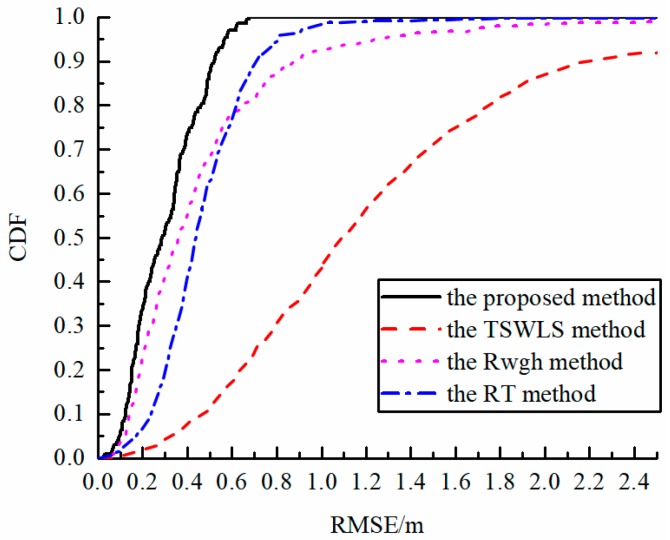
CDF curve of the algorithm.

Figure 6 compares the CDF curves of four algorithms when σ is 0.2 m, where there were 2 NLOS base stations, and the maximum possible error of NLOS was 3 m. According to the results in the figure, it can be seen that the algorithm error proposed in this paper is significantly smaller than other algorithms. In addition, the proposed algorithm is smooth when the error value is less than 0.6 m. This shows that under this simulation condition, the maximum localization error of the proposed algorithm is less than 0.6 m, and it is unlikely to cause misjudgment in the application.

## 4. Field Test in Substation

### 4.1. Test Arrangement

**Figure 7:**
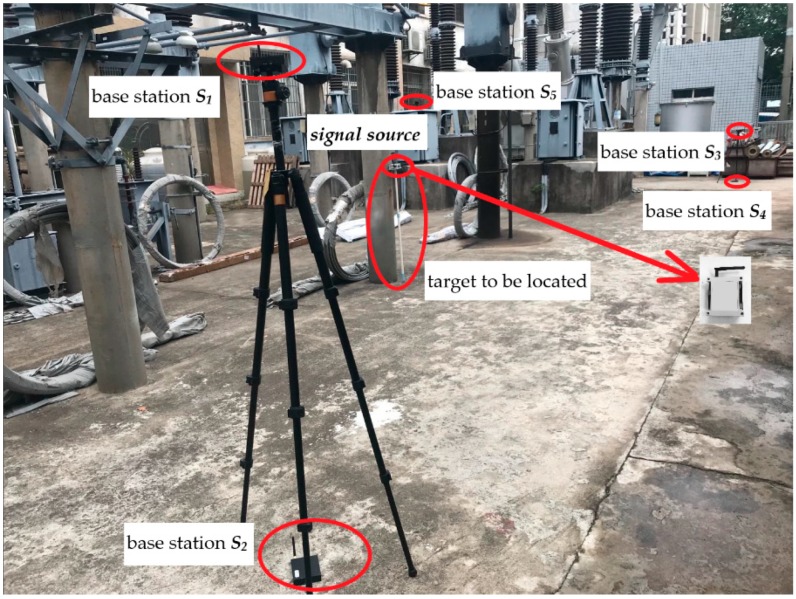
Test substation site layout.

**Figure 8:**
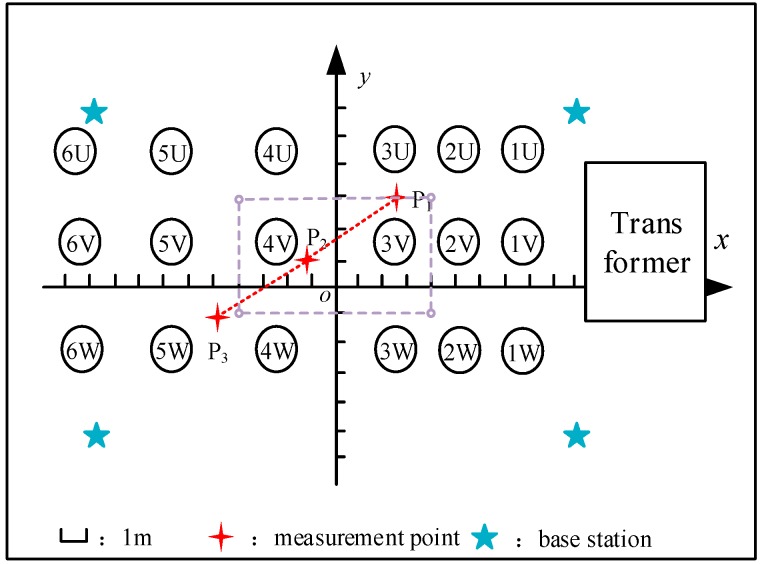
Schematic diagram of substation experimental layout.

To verify the simulation results, a localization test was carried out in a 220 kV substation. The photo of the substation site layout is shown in Figure 7. Figure 8 is a plain view of the substation test arrangement. In Figure 8, 1 is a lightning arrester; 2 is a voltage transformer; 3 is a circuit breaker; 4 is a current transformer; 5 is an isolating switch; 6 is a coupling capacitor, and U, V, and W represent three phases of a high voltage device. In the space rectangular coordinate system established in the substation, the unit of coordinates is meters. The eight localization base stations had the coordinates ***S*_1_** (−9.5, −5.5, 1.8), ***S*_2_** (−9.5, −5.5, 0), ***S*_3_** (9.5, −5.5, 1.8), ***S*_4_** (9.5, −5.5, 0), ***S*_5_** (9.5, 5.5, 1.8), ***S*_6_** (9.5, 5.5, 0), ***S*_7_** (−9.5, 5.5, 1.8), ***S*_8_** (−9.5, 5.5, 0). The UWB measurement module was arranged at the corresponding location to obtain relevant measurement information. At the same time, the tag for the signal source was fixed on a rigid long rod to serve as a localization target, and the height of the tag was 1.5 m. In this paper, two paths were designed in the experiment. One was a straight path and the other was a rectangular path. For the straight path, the localization target carrying the tag was moved manually from measuring point P_1_ (2.5, 3, 1.5) to measuring point P_2_ (−1.1, 0.76, 1.5) and finally to measuring point P_3_ (−4.8, −1.6, 1.5). For the rectangle path, the four vertices of the path were (−4, 3, 1.5), (4, 3, 1.5), (4, −1, 1.5), (−4, −1, 1.5), respectively. The target moves along a rectangular path determined by these four vertices.

### 4.2. Test Result and Discussion

The time difference measured between the base station in the field is calculated by using the probability distribution-based localization algorithm, the RWGH localization algorithm, the RT algorithm, and the TSWLS algorithm, respectively. To better verify the superiority of the proposed algorithm, this section also adds the localization trajectory of the TSWLS localization algorithm improved by the PF [26] method, and compares it with each algorithm.

**Figure 9:**
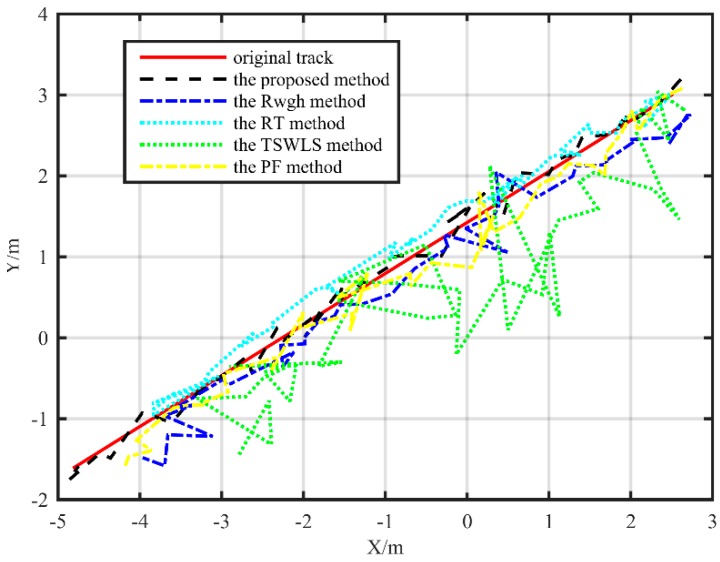
Substation test localization trajectory map under a straight path (XY plane).

**Figure 10:**
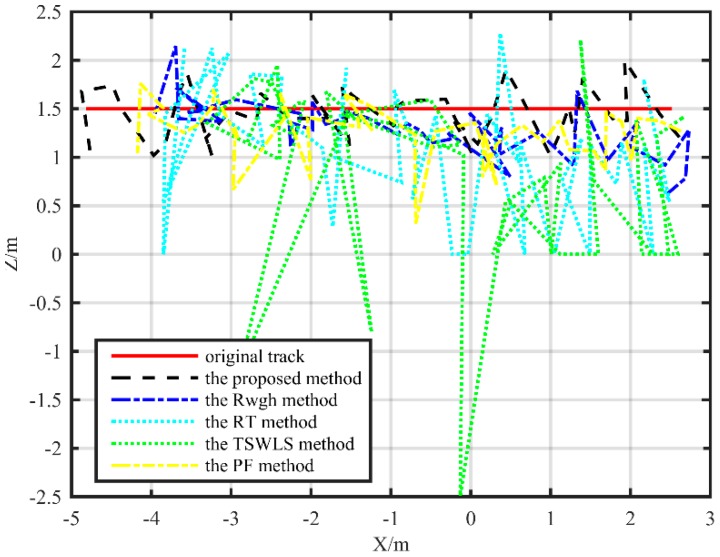
Substation test localization trajectory map under a straight path (XZ plane).

The localization track of the straight path is shown in Figure 9 and Figure 10, from which it can be seen that the localization error of the proposed method is significantly smaller than other localization methods. In the XY plane, in addition to the TSWLS algorithm, the localization trajectories of the other four localization algorithms are basically distributed next to the actual trajectory. The algorithm proposed in this paper is more precise than the RWGH algorithm, the RT algorithm, and the PF method. In the XZ plane, the localization algorithm proposed in the paper has far smaller error in height localization than the RWGH algorithm and the PF algorithm. The RT algorithm has a large error. When the target moved along a straight path, the RMSE value of the localization value and the real value of the proposed algorithm was 0.25 m, the RMSE obtained by the PF algorithm was 0.34 m, the RMSE of the RWGH algorithm and the real value was 0.52 m, and the RMSE obtained by the RT algorithm was 1.09 m. The RMSE obtained by the TSWLS algorithm was 1.41 m. From the results, we can see that under the linear trajectory, the proposed algorithm has the best localization performance.

**Figure 11:**
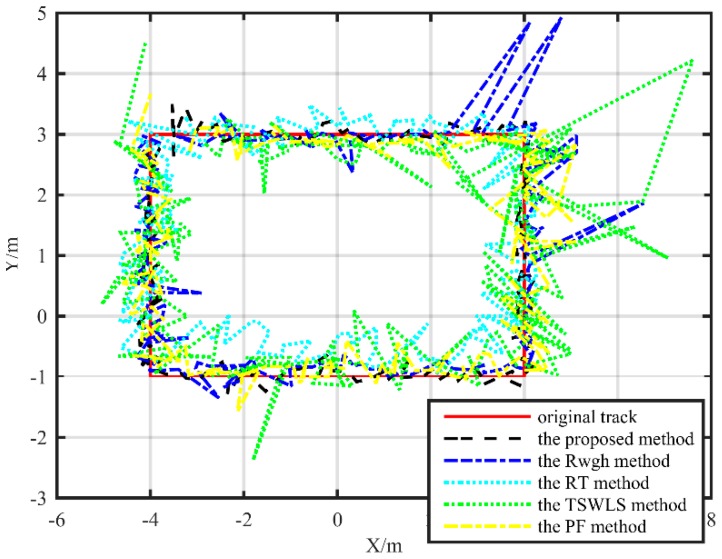
Substation test localization trajectory map under a rectangle path (XY plane).

**Figure 12:**
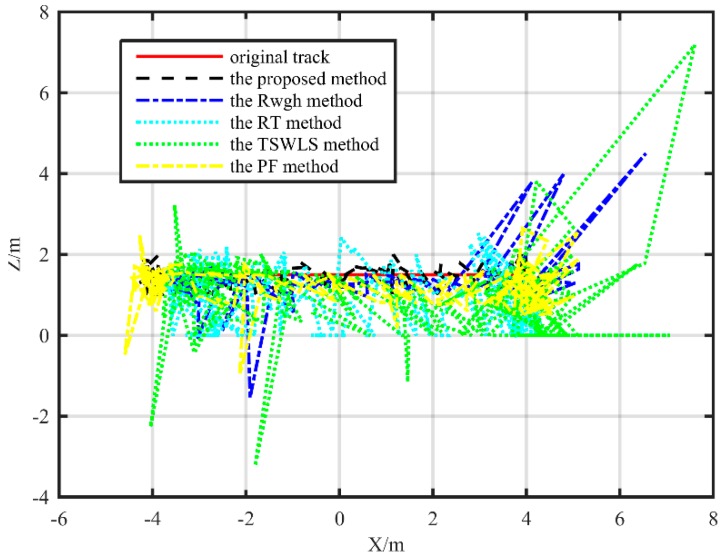
Substation test localization trajectory map under a rectangle path (XZ plane).

The localization track of the rectangle path is shown in Figure 11 and Figure 12 The localization accuracy of the proposed method is obviously better than other methods in both XY plane and XZ plane. When the target moved along a rectangle path, the RMSE value of the localization value and the real value of the proposed algorithm was 0.28 m, the RMSE obtained by the PF algorithm was 0.55 m, the RMSE of the RWGH algorithm and the real value was 0.59 m, and the RMSE obtained by the RT algorithm was 0.94 m. The RMSE obtained by the TSWLS algorithm was 1.38 m. The localization error of the PF localization algorithm under the rectangular trajectory is slightly increased. This is because the motion state changes at the turning point of the moving target, and the correction effect of the PF localization algorithm on the localization trajectory will decrease. Under the rectangular trajectory, the localization error of the proposed localization algorithm is still the lowest among these five algorithms.

The RMSE of the localization results and the true values of each of the algorithms under the scenarios of two different paths is shown in Table 2. From Table 2, we can see that in the case of NLOS, TSWLS cannot guarantee the accuracy of localization result. From Figure 9 to Figure 12, we can see that the localization result of TSWLS in this environment is not stable. From the results, we can also see that the PF can effectively improve the initial localization result of the TSWLS algorithm. The correction effect of the PF algorithm on the linear trajectory is better than the correction effect on the rectangular trajectory. This is because the moving state of the moving target changes significantly at the corner, and in the localization system with only the distance sensor, the motion state of the object itself cannot be corrected very well. Finally, it can be seen from Table 2 that the localization accuracy of the proposed algorithm in both paths does not change significantly, and the RMSE values are less than 30 cm, which is the minimum of among all the algorithms. When the object moves along a linear path, the localization error of the proposed algorithm is only 0.09 m lower than the PF localization method. However, on the rectangular trajectory, the localization error of the proposed algorithm is reduced by 0.27 m compared with the PF localization algorithm. This is because the algorithm proposed in the paper does not rely on other state information of the target to correct the localization result. The algorithm proposed in this paper reduces the influence of measurement error on the localization result based on the probability distribution function of the initial localization result of the group, so it is less affected by the moving trajectory of the target.

The average time consumption of an algorithm for a single point in the localization process is shown in Table 3, from which we can see that the algorithm mentioned in the paper takes the second longest time of these five localization algorithms, but it did not exceed 0.1 s. The localization target moving speed in the substation is not too fast; although the algorithm proposed in the paper is not as efficient as the existing algorithm, it can still meet the requirement in practical application. How to further improve the computational efficiency of the proposed algorithm is an area for improvement in follow-up studies.

To further analyze the influence of measurement error on the localization accuracy of the proposed algorithm and the RWGH algorithm, the below analyzes the localization results of P_1_, P_2_, and P_3_.

Table 4 lists the time difference among the time when the tag signal is at P_1_, P_2_, and P_3_, and the difference between the time difference measurement and the real-time difference. T_i1_ in the table indicates the time difference between the arrival of the signal at the i-th base station and the first base station.

As can be seen from Table 4, the time measurement error, when the base station is in the LOS situation, generally does not exceed 0.5 ns. The closer the measurement distance, the smaller the measurement error. When the localization base station is occluded, the measurement error changes depending the severity of the occlusion. Localization base stations 5 and 6 may be taken as an example for further analysis. These two base stations were in front of a lightning arrester, and localization base station 5 was placed on the ground, so that the occlusion is severe. However, since localization base station 6 was placed high above ground, the occlusion is not obvious when the measurement point was far away, and the measurement error did not change significantly.

The data in Table 4 was made available to apply the RWGH algorithm and to apply the proposed algorithm for localization. The results are shown in Table 5. The accuracy of the algorithm is represented by the RMSE value of the localization result and the actual result. The unit of the coordinates and the RMSE value in Table 5 are in meters. It can be seen from Table 5 that the localization algorithm proposed in this paper is more stable than the RWGH algorithm, especially when the NLOS measurement error is serious. Taking P_1_ as an example, the localization performance of the proposed method is significantly better than the existing algorithm. At the same time, among the three localization points, the final localization errors of the localization points P_2_ and P_3_ are small. This is because the positions of P_2_ and P_3_ are relatively empty, the error of the time difference measurement from the point to each base station is small.

Combined with the data of the field test, the accuracy of the time difference measurement has a great influence on the localization result. The localization algorithm proposed in this paper can effectively reduce the influence of measurement error on localization error, and each single coordinate error fell within 30 cm. In the actual application in the substation, in addition to considering the placement of the localization base station and improving its measurement performance, the localization method proposed in this paper can effectively reduce the influence of measurement error and make the localization result more reliable.

## 5. Conclusions

This paper proposes a new localization method based on TSWLS and probability distribution function. The proposed method obtains multiple sets of initial localization results using the two-step weighted least-squares method, and constructs the probability distribution function of the target by using the initial localization results. The final localization result is obtained by solving the objective function. This localization method reduces the influence of measurement error on the localization result, and can effectively improve the stability and effectiveness of the localization algorithm. For the traditional LS localization, the localization result obtained by the data with more LOS measurements will be closer to the actual coordinates of the target, and will be regularly distributed around the actual coordinates. The initial localization results obtained by the data with more NLOS measurements will have large deviations, which show no regularity. By constructing the probability distribution function, the initial localization results that are greatly affected by the NLOS error can be effectively eliminated. This can help solve the shortcoming that the traditional least-squares-based localization method is greatly affected by the NLOS error. At the same time, through the simple base station screening algorithm, the algorithm proposed in the paper has better computational efficiency.

The results of the simulation analysis and the field test show that the average localization error is reduced by more than one meter compared with the TSWLS method when the measurement error is large. Compared with the RWGH algorithms and the RT method, the proposed algorithm has improved the localization accuracy by more than 50%. Compared with the PF method, the proposed method also has better performance. When the object moves along a linear path, the localization error of the proposed algorithm is 0.09 m lower than the PF localization method. However, on the rectangular trajectory, the localization error of the proposed algorithm is reduced by 0.27 m compared with the PF localization algorithm. This method can keep the coordinate error within 30 cm in the substation environment. This proves that the localization method proposed in this paper can achieve precise localization of targets in substation environments with only the ranging sensors, although the computational efficiency of the proposed algorithm is not as efficient as some existing algorithms. The average time required for a single-point calculation using the proposed algorithm is less than 0.1 s on an ordinary PC. It can meet the basic requirements of the mobile target location in the substation.

## Figures and Tables

**Table 1:** Simulation parameter setting.

	σ/m	*B*/m	Number of NLOS Base Stations
Simulation 1	variable	0	0
Simulation 2	0.2	variable	2
Simulation 3	0.2	3	variable
Simulation 4	0.2	3	2

**Table 2:** The RMSE of different localization algorithms under the two paths.

	The Proposed Method	The RWGH Method	The RT Method	The TSWLS Method	The PF Method
straight path	0.25 m	0.52 m	1.09 m	1.41 m	0.34 m
rectangle path	0.28 m	0.59 m	0.94 m	1.38 m	0.55 m

**Table 3:** The average time taken of different localization algorithms for single point.

	The Proposed Method	The RWGH Method	The RT Method	The TSWLS Method	The PF Method
time taken	0.094 s	0.058 s	0.026 s	4.69 × 10^−4^ s	0.126 s

**Table 4:** Measurement values of arrival time difference of each measurement point and its error.

Time Difference	P_1_		P_2_		P_3_	
	Measurements	Error	Measurements	Error	Measurements	Error
T_21_	−0.24 ns	0.15 ns	−0.17 ns	0.17 ns	−0.66 ns	−0.08 ns
T_31_	−7.90 ns	4.33 ns	6.87 ns	0.81 ns	29.28 ns	0.58 ns
T_41_	−9.84 ns	2.71 ns	6.42 ns	0.65 ns	28.10 ns	−0.35 ns
T_51_	−17.61 ns	6.39 ns	7.26 ns	3.51 ns	28.18 ns	4.31 ns
T_61_	−21.65 ns	2.83 ns	3.86 ns	0.47 ns	31.38 ns	0.89 ns
T_71_	−8.22 ns	−0.11 ns	−2.42 ns	0.32 ns	8.10 ns	0.24 ns
T_81_	−8.72 ns	−0.32 ns	−3.29 ns	−0.19 ns	7.63 ns	0.19 ns

**Table 5:** Comparison of localization results of two localization methods.

Actual	The Proposed Method		The RWGH Method	
Coordinates/m	Result/m	RMSE/m	Result/m	RMSE/m
P_1_ (2.5,3,1.5)	(2.63,3.18,1.28)	0.313	(2.44,2.56,0.65)	0.960
P_2_ (−1.1,0.76,1.5)	(−0.99,0.87,1.34)	0.223	(−0.89,0.63,1.28)	0.339
P_3_ (−4.8,−1.6,1.5)	(−4.68,−1.68,1.66)	0.219	(−4.96,−1.74,1.32)	0.278
